# Serological Response in Lung Transplant Recipients after Two Doses of SARS-CoV-2 mRNA Vaccines

**DOI:** 10.3390/vaccines9070708

**Published:** 2021-06-30

**Authors:** Madhusudhanan Narasimhan, Lenin Mahimainathan, Andrew E Clark, Amena Usmani, Jing Cao, Ellen Araj, Fernando Torres, Ravi Sarode, Vaidehi Kaza, Chantale Lacelle, Alagarraju Muthukumar

**Affiliations:** 1 Department of Pathology, University of Texas Southwestern Medical Center, Dallas, TX 75390, USA; madhusudhanan.narasimhan@utsouthwestern.edu (M.N.); lenin.mahimainathan@utsouthwestern.edu (L.M.); andrew.clark@utsouthwestern.edu (A.E.C.); amena.usmani@UTSouthwestern.edu (A.U.); jing.cao2@UTSouthwestern.edu (J.C.); ellen.araj@utsouthwestern.edu (E.A.); ravi.sarode@utsouthwestern.edu (R.S.); 2 Department of Internal Medicine, University of Texas Southwestern Medical Center, Dallas, TX 75390, USA; fernando.torres@UTSouthwestern.edu (F.T.); vaidehi.kaza@UTSouthwestern.edu (V.K.)

**Keywords:** lung transplantation, SARS-CoV-2, IgG, IgM, Spike, nucleocapsid, COVID-19 mRNA vaccines, two doses, serological response

## Abstract

Background: Lung-transplant (LT) recipients are at high risk for COVID-19 due to immunosuppression and respiratory tropism of SARS-CoV-2. The information on the effect of COVID-19 mRNA vaccines to elicit immunogenic responses after a two-dose (2D) regimen in LT recipients is sparse. Thus, we assessed the effect of Pfizer-BioNTech and Moderna mRNA vaccines’ 2D regimen on anti-spike responses in immunocompromised LT recipients. Methods: We utilized serum samples from LT recipients vaccinated for SARS-CoV-2 with 2D of either the Pfizer-BioNTech or Moderna vaccines and 2D-vaccinated naïve (non-transplanted and non-exposed to COVID-19) group. Antibody responses were assessed using the FDA-approved SARS-CoV-2 anti-nucleocapsid protein IgG assay (IgG_NC_), the SARS-CoV-2 anti-spike protein IgM assay (IgM_SP_), and the SARS-CoV-2 anti-spike protein IgG II assay (IgG_SP_). CD4+ T-cell activity was assessed as a marker of immune competence using the ImmuKnow^®^ assay. Results: About 25% (18/73) of SARS-CoV-2 uninfected-LT patients generated a positive spike-IgG response following 2D of vaccines, with 36% (9/25) in the Moderna cohort and only 19% (9/48) in the Pfizer cohort. 2D in LT patients elicited a significantly lesser median IgG_SP_ response (1.7 AU/mL, 95% CI: 0.6–7.5 AU/mL) compared to non-transplanted, uninfected naïve subjects (14,209 AU/mL, 95% CI: 11,261–18,836 AU/mL; *p* < 0.0001). In LT patients, the Moderna-evoked seropositivity trend was higher than Pfizer. Conclusion: 2D COVID-19 vaccination elicits a dampened serological response in LT patients. Whether assessing other arms of host immunity combined with a higher vaccine dose can better capture and elicit improved immunogenicity in this immunocompromised population warrants investigation.

## 1. Introduction

Lung-transplant (LT) recipients are at high risk for severe COVID-19 due to immunosuppression (IS) and respiratory tropism for SARS-CoV-2. Although SARS-CoV-2 vaccine-related immunogenicity in solid organ transplant (SOT) has emerged [1], the impact of a two-dose vaccine regimen on the quality of humoral immunological response in LT recipients has scarcely been reported. Moreover, published results of SOT patients comprising the LT sub-group used a small number of patients with an assessment of immunogenicity of either one of the mRNA vaccines [2,3]. Importantly, still precise immune correlates of protection related to vaccination remain uncertain in this population [2,3,4].

We and others have demonstrated that the serological assessment of IgG and IgM against SARS-CoV-2 nucleocapsid (NC) and spike (SP) proteins is reliable in differentiating humoral responses to infection versus vaccination [2,3,4,5,6]. A recent study has evaluated a conservative IgG Spike-Receptor Binding Domain (S-RBD) level (Abbott assay) as a surrogate measure of antibody neutralization and identified that at or above 4160 AU/mL, the IgG (S-RBD) titer estimates consistently corresponded to a 0.95 probability of the plaque reduction neutralization test (PRNT; the gold standard) proportion at a 50-stringency threshold for 1/250 dilution.

Given this knowledge of conservative threshold and the need to understand the impact of vaccine regimens on humoral immunity to optimize the COVID-19 immunization in this immunocompromised cohort, we sought to address the effect of Pfizer-BioNTech and Moderna mRNA vaccines’ two-dose (2D) regimen on humoral responses in immunocompromised LT recipients, using a combination of an orthogonal approach comprising of IgG_NC_, SP-specific-IgG (IgG_SP_), and IgM (IgM_SP_) analysis.

## 2. Materials and Methods

LT recipients and naïve (non-transplanted and non-exposed to COVID-19) group vaccinated for SARS-CoV-2 with 2D of either the Pfizer-BioNTech (New York, NY, USA) or Moderna vaccines (Cambridge, MA, USA) between 15 December 2020 and 19 March 2021 were included in this study. The University of Texas Southwestern Medical Center’s institutional review board waiver of consent approval was obtained for the use of excess, remnant samples available from these patients in our clinical diagnostic laboratory.

Antibody responses were semi-quantitatively assessed using serum samples analyzed on the Alinity i platform (Abbott Laboratories, Abbott Park, IL, USA) using the FDA-approved SARS-CoV-2 anti-nucleocapsid protein IgG assay (IgG_NC_), the SARS-CoV-2 anti-spike protein IgM assay (IgM_SP_), or the SARS-CoV-2 anti-spike protein IgG II assay (IgG_SP_), as previously described. Two Index values of ≥1.4 (IgG_NC_), ≥1.0 (IgM_SP_), and ≥50 AU/mL (IgG_SP_) were interpreted as positive per the manufacturer’s recommended threshold. IgG_NC_ positivity informs natural SARS-CoV-2 infection, while IgG_SP_/IgM_SP_ positivity strongly correlates with the emergence of natural or vaccine-driven neutralizing immunity [5,7]. CD4+ T-cell activity was assessed as a marker of immune competence using the ImmuKnow^®^ assay (Viracor-IBT, Lee’s Summit, MO, USA). Results were interpreted as either low, moderately, or strongly correlating with manufacturer-established Adenosine tri phosphate (ATP) ranges of ≤225, 226–524, and ≥525 ng/mL, respectively.

A two-tailed unpaired t-test was performed to assess statistical significance using GraphPad Prism 9.1.0. A value of *p* < 0.05 was considered statistically significant.

## 3. Results

This study included 73 LT recipients and their samples were collected, and analyzed between 1 February 2021 and 19 March 2021 (Table 1). The median age was 65 years (Interquartile range (IQR), (53.5–69.5 years)), 74% were male. A total of 66% received the Pfizer vaccine, and 34% received the Moderna formulation, with the exception of one recipient who was not on anti-metabolite. Further, two were on cyclosporine, and one was on a combination of low-dose tacrolimus and sirolimus. The median time since transplant surgery (TSTS) for the study participants was 40 months (IQR, 44 (19–63 months)). IgG_NC_ assessment confirmed the absence of any previous silent or asymptomatic COVID-19 infection in naïve, non-transplant cohorts with one previously infected case in the LT cohort.

**Figure 1:**
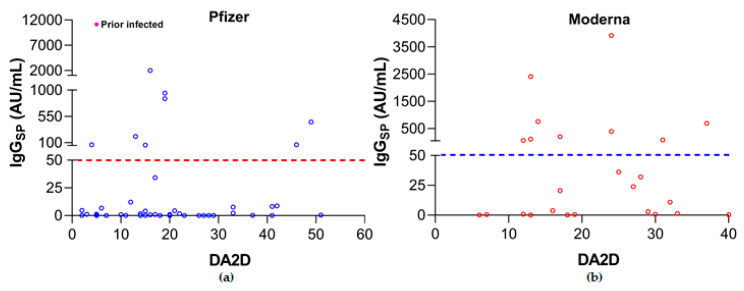
Frequency distribution of IgG_SP_ versus days after the second dose of COVID-19 vaccines in LT recipients (**a**) BNT162b2 (Pfizer-BioNTech); (**b**) mRNA-1273 (Moderna). DA2D, days after the second dose of COVID-19 vaccines; prior-infected, pink-filled circle (Figure 1a); broken red and blue lines, IgG_SP_ serology assay’s manufacturer-recommended positive cut-off value.

Only 25% (18/73) of LT recipients demonstrated IgG_SP_-positivity following 2D of mRNA vaccines. At a median of 17.5 days after the 2D of the Pfizer vaccine, only 19% (9/48) displayed positive IgG_SP_ levels. In contrast, at a median time of 19 days after 2D of Moderna-mRNA vaccine, 36% (9/25) had IgG_SP_-positivity (Table 1; Figure 1). In addition, when applying the conservative threshold of >4160 AU/mL, an estimate shown to have 95% concordance with plaque reduction neutralization test (PRNT) assay-based neutralizing antibody titer determination [8], only one Pfizer subject had values above the neutralizing threshold (11,149 AU/mL; Figure 1a; pink filled circle), however, the patient had previously been infected by SARS-CoV-2. This data may mean an inadequate protective response in LT-vaccinated subjects.

In total, 56 of the 73 LT recipients had Cylex ImmuKnow assay values measured. The ImmuKnow Immune Cell Function Assay is an FDA-approved test for detecting cell-mediated immunity in an immunosuppressed patient. The test measures the CD4 T-cell activation by quantifying the amount of intracellular ATP synthesis derived from the circulating CD4 T-cells following a non-specific mitogenic phytohemagglutinin (PHA) stimulation. The released ATP was then processed through a luminometer to calculate the numeric result, and based on these numeric ATP values, the CD4 T-cell response was stratified as low (ATP < 225 ng/mL), moderate (ATP 226–524 ng/mL), and strong (ATP > 525 ng/mL) [9]. The Cylex ImmuKnow assay’s successful utility as an adjuvant immunosuppression monitoring technique is demonstrated in various solid organ transplant conditions, including but not limited to lung, liver, and kidney patients [10,11,12]. In particular, the ImmuKnow assay has been reported as a more stable method to apply for targeted monitoring and/or assessing immunosuppression of a ‘low responders’ pool, who are past 6 months of their transplant surgery. Notably, it is regarded to be relatively less sensitive as a screening test during the initial 6 months after transplant owing to a consistent pattern of reductions in T-cell numbers [13]. The Moderna vaccine was found to elicit positive IgG_SP_ responses in 44% (4/9) of the patients having moderate, and 50% (1/2) of the patients having strong Cylex assay values. In contrast, the Pfizer vaccine elicited a positive IgG_SP_ response only in 18% (3/17) of patients with a moderate Cylex response and none (0/6) in patients with strong Cylex ImmuKnow levels (Table 2). For either vaccine, the Cylex assay did not allow to predict which patients may yield a better antibody response for IgG_SP_, IgM_SP_, or IgG_NC_.

**Figure 2:**
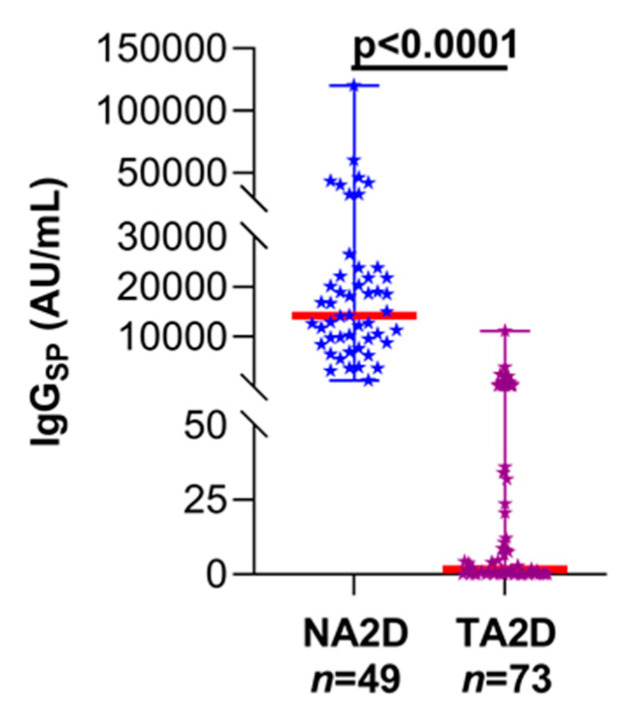
IgG_SP_ levels in naïve subjects and LT patients after 2 doses of COVID-19 vaccine. NA2D, naïve subjects (non-transplanted and not had previous SARS-CoV-2 infection) after 2 doses of vaccine; TA2D, LT patients after 2 doses of vaccine; thick red line, median value.

**Figure 3:**
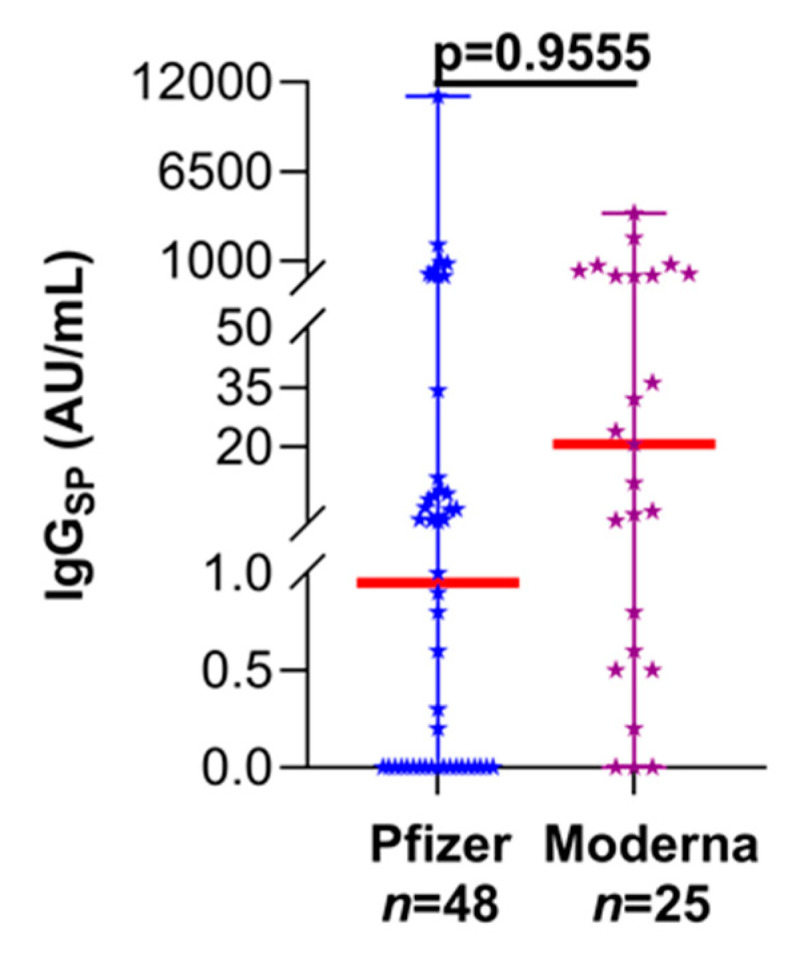
Evaluation of immunogenicity following the double-dose Pfizer and Moderna vaccine regimen in LT recipients. Thick red line, median value; median (95% CI) (Pfizer—0.9 (0.0–4.1); Moderna—20.6 (0.8–80.2)).

Comparison of SARS-CoV-2 specific antibody responses following a 2D regimen of mRNA vaccine clearly illustrated that the immunosuppressed LT recipients had significantly decreased median IgG_SP_ levels (1.7 AU/mL, 95% CI: 0.6–7.5 AU/mL) when compared to the non-transplant, naïve (never SARS-CoV-2 infected) participants (14,209 AU/mL, 95% CI: 11,261–18,836 AU/mL; *p* < 0.0001; Figure 2). Furthermore, albeit statistically non-significant, a lower circulating IgG_SP_ trend with the 2D Pfizer vaccine was noted than the 2D Moderna formulation among LT patients (Figure 3; 23-fold, *p* = 0.9555).

## 4. Discussion

Immunization against SARS-CoV-2 has proven effective, with the potential to restrain viral propagation and prevent severe illnesses in the general population. However, studies evaluating the effectiveness of the COVID-19 vaccine in immunocompromised subgroups, including LT recipients, are a work in progress. In this study, we estimated the extent to which the Pfizer and Moderna COVID-19 vaccines following the 2D regimen evoked SARS-CoV-2-specific antibody responses in a cohort almost twice the size of previous studies with LT patients [1,2,3]. Our data showed that the majority of the LT recipients demonstrated sub-optimal immunogenicity, with only 25% of the participants mounted appreciable anti-spike antibody responses. In addition, the ImmuKnow Cylex assay-based results suggested that the immune-cell-function-based stratification does not predict an antibody response to the vaccines. However, irrespective of the immune cell function, the Moderna formulation generated a trend towards a more robust antibody response than the Pfizer vaccine in immunosuppressed-LT patients, indicating an enhanced protective immunity. The principle of generating a mRNA vaccine that encodes a SARS-CoV-2 spike protein that is stabilized in the prefusion conformation along with the larger dose of 100 µg in the Moderna versus the 30 µg in the Pfizer preparation could explain the differential antibody response obtained when comparing the two vaccines [14,15]. Similar findings were reported recently [1], with the Moderna group developing an antibody response at 69% vs. 31% in the Pfizer group.

Importantly, our data indicated that receiving two doses of vaccine does not mean assured protection against a COVID-19 infection for the majority of LT patients, as the majority exhibited either nil or negligible IgG_SP_ responses. Additionally, when compared with the naïve vaccinated (immunocompetent) group, the vaccinated LT patients without prior infection that mounted an antibody response appeared to generate a response not comparable to that of a neutralizing antibody titer, as derived from the application of the conservative threshold of >4160 AU/mL [7]. The observed low concordance of IgG_SP_ serology data with the conservative neutralizing threshold value may imply an inadequate protective response. While this correlation approach may serve as a proxy for neutralization and has been previously used to obtain a simple immunological meaning [16,17], its clinical utility warrants future rigorous studies. At the same time, this immune paresis in LT cohorts following vaccination does not mean they lack complete protection, as B-cell negative X-linked Agammaglobulinemia patients have been recently shown to benefit from vaccination due to robust cellular immunity and/or T-cell responses [18]. How and why such preferential manipulation of immune response occurs is although beyond the scope of the current study; we speculate that there is a possibility of cellular immunity being relatively more resistant than the humoral arm for certain immunosuppressive drugs, as reported for the imidazole-4-carboxamide, 5-(3,3-dimethyl-1-triazeno; DTIC) that is used in the palliation of malignant melanoma [19]. Nevertheless, when there is no antibody response, as noted in the vaccinated LT patients, evidence and data from the non-COVID-19 context support that the doubling of the vaccine dose could be an attractive strategy to increase the immunogenicity [20]. However, the theoretical risk of vaccines and alloimmunity and rejection remains, although not a consistent observation so far.

Notably, the current study does not include the assessment of immunogenicity that may result from the administration of FDA-authorized adenovirus (Adv)-based replication-incompetent vector vaccines due to a lack of Adv vaccines-immunized subjects at the time of communication. However, early reports from other laboratories indicated that both the immunocompetent controls and the immunocompromised transplant patients immunized with Adv-based vaccines showed an impaired humoral immune response [21]. Contrastingly, the same study showed that Adv-based vaccines revealed a superior cellular immune response (triple (IFNγ, IL-2, and TNFα) cytokine expressing CD4 T cells) compared to mRNA vaccines and suggested the need for a combined analysis of humoral and cellular immunity to identify relatively ‘true’ vaccine responders in both the immunocompetent and immunocompromised individuals. Future studies must expand on the current finding with respect to assessing the humoral and cellular immune response involving CD4 and CD8 T cells along with the neutralization capacity in LT patients relative to age- and vaccine-matched controls.

Limitations include the small sample size, lack of demographic data in the non-transplant group, absence of serial measurements after vaccination, and shorter time for follow-up. Despite these limitations, we demonstrated that a two-dose COVID-19 mRNA vaccine elicits a serological response, though not in a majority of LT patients. Further studies are needed to understand the efficacy and longevity of vaccine-derived COVID-19 immunity in this vulnerable population. Since most lung transplant recipients are maintained at a higher level of immunosuppression compared to other solid organ transplant recipients, larger studies with a longer duration of follow-up are needed to confirm our preliminary findings of antibody response after completing the SARS-CoV-2 mRNA vaccination.

## 5. Conclusions

In sum, the current standard two-dose mRNA vaccine regimen resulted in a restrained SARS-CoV-2-specific antibody response in LT recipients. Further, a relatively higher anti-spike response was observed in Moderna compared to Pfizer-vaccinated LT patients, implying the potential benefit of a higher antigenic dose (as in the case of Moderna) with possible room for an additional vaccine booster in these selected candidates. Importantly, while arguing in favor of vaccinating these immune-suppressed LT cohorts, our data also cautions that, due to immune paresis, it is imperative that these candidates continue to strictly follow the hand hygiene protocols, masking, and physical distancing regardless of immunization.

## Figures and Tables

**Table 1:** Demographic and clinical characteristics of study participants stratified by spike-specific IgG (IgG_SP_) antibody response after two-dose-COVID-19 vaccination.

Information	IgG_SP_		
	Total *n* (%)	Positive *n* (%)	Negative *n* (%)
2D-vaccine-administered total LT subjects	73 (100)		
**Age (Years)**			
20–39	10 (14)	2 (20)	8 (80)
40–59	17 (23)	9 (53)	8 (47)
≥60	46 (63)	7 (15)	39 (85)
**Sex**			
Female	26 (36)	7 (27)	19 (73)
Male	47 (64)	11 (23)	36 (77)
**Race**			
White	55 (75)	13 (24)	42 (76)
Non-White	18 (25)	5 (28)	13 (72)
**TSTS ^a^ (Months)**			
1–50	47 (64)	12 (26)	35 (74)
51–100	23 (32)	5 (22)	18 (78)
>100	3 (4)	1 (33)	2 (67)
**DMR ^b^**			
ON anti-metabolite ^c^	72 (99)	18 (25)	54 (75)
OFF anti-metabolite	1 (1)	0	1 (100)
**Vaccine**			
BNT162b2 (Pfizer-BioNTech)	48 (66)	9 (19)	39 (81)
mRNA-1273 (Moderna)	25 (34)	9 (36)	16 (64)

^a^, time since transplant surgery (TSTS); ^b^, drug maintenance regimen; ^c^, antimetabolite including mycophenolate mofetil (MMF), mycophenolate sodium, or azathioprine.

**Table 2:** IgM_SP_, IgG_NC_, and IgG_SP_ responses following a double-dose COVID-19 vaccine regimen in lung transplant patients stratified by Cylex ImmuKnow assay levels.

Information	*n* (%)	Cylex ImmuKnow Assay Levels		
		Low *n* (%)	Moderate *n* (%)	Strong *n* (%)
**2D-vaccine-administered LT subjects that had Cylex results**	56/73 (77)	22/56 (39.3)	26/56 (46.4)	8/56 (14.3)
	**IgG_SP_ serology results**			
BNT162b2 (Pfizer-BioNTech)	+	3/15 (20)	3/17 (18)	0/6 (--)
	−	12/15 (80)	14/17 (82)	6/6 (100)
mRNA-1273 (Moderna)	+	1/7 (14)	4/9 (44)	1/2 (50)
	−	6/7 (86)	5/9 (56)	1/2 (50)
	**IgM_SP_ serology results**			
BNT162b2 (Pfizer-BioNTech)	+	1/15 (7)	1/17 (6)	0/6 (--)
	−	14/15 (93)	16/17 (94)	6/6 (100)
mRNA-1273 (Moderna)	+	0/7 (--)	0/9 (--)	0/2 (--)
	−	7/7 (100)	9/9 (100)	2/2 (100)
	**IgG_NC_ serology results**			
BNT162b2 (Pfizer-BioNTech)	+	0/15 (--)	1/17 (6)	0/6 (--)
	−	15/15 (100)	16/17 (94)	6/6 (100)
mRNA-1273 (Moderna)	+	0/7 (--)	0/9 (--)	0/2 (--)
	−	7/7 (100)	9/9 (100)	2/2 (100)

+ and −, serology assay’s positive and negative results based on the manufacturer-recommended cut-off value.

## Data Availability

Due to ethical reasons, the data are not publicly available. However, upon request, the data presented in this study can be shared.
